# Using the One-Lung Method to Link p38 to Pro-Inflammatory Gene Expression during Overventilation in C57BL/6 and BALB/c Mice

**DOI:** 10.1371/journal.pone.0041464

**Published:** 2012-07-24

**Authors:** Stephanie Siegl, Stefan Uhlig

**Affiliations:** Institute of Pharmacology and Toxicology, Medical Faculty, RWTH Aachen University, Aachen, Germany; University of Tübingen, Germany

## Abstract

**Introduction:**

The mechanisms of ventilator-induced lung injury (VILI), including the role of MAP kinases, are frequently studied in different mouse strains. A useful model for such studies is the isolated perfused mouse lung. As a further development we present the one-lung method that permits to continue perfusion and ventilation of the right lung after removal of the left lung. This method was used to compare the effect of high pressure ventilation (HPV) on pro-inflammatory signaling events in two widely used mouse strains (C57BL/6, BALB/c) and to further define the role of p38 in VILI.

**Methods:**

Lungs were perfused and ventilated for 30 min under control conditions before they were randomized to low (8 cm H_2_O) or high (25 cm H_2_O) pressure ventilation (HPV) for 210 min, with the left lung being removed after 180 min. In the left lung we measured the phosphorylation of p38, JNK, ERK and Akt kinase, and in the right lung gene expression and protein concentrations of Il1b, Il6, Tnf, Cxcl1, Cxcl2, and Areg.

**Results:**

Lung mechanics and kinase activation were similar in both mouse strains. HPV increased all genes (except *Tnf* in BALB/c) and all mediators in both strains. The gene expression of mRNA for *Il1b, Il6, Cxcl1* and *Cxcl2* was higher in BALB/c mice. Backward regression of the kinase data at t = 180 min with the gene and protein expression data at t = 240 min suggested that p38 controls HPV-induced gene expression, but not protein production. This hypothesis was confirmed in experiments with the p38-kinase inhibitor SB203580.

**Conclusions:**

The one-lung method is useful for mechanistic studies in the lungs. While C57BL/6 show diminished pro-inflammatory responses during HPV, lung mechanics and mechanotransduction processes appear to be similar in both mouse strains. Finally, the one-lung method allowed us to link p38 to gene expression during VILI.

## Introduction

Patients with acute respiratory distress syndrome (ARDS) require mechanical ventilation and are at risk for ventilator-induced lung injury (VILI). High strain and stress can result in physical injury termed barotrauma/volutrauma and atelectotrauma as well as in exaggerated inflammatory responses termed biotrauma [1]. In the ARDSNet study in 2000 [2] that set the current standard of ventilation of ARDS patients mortality correlated with inflammation, but not with barotrauma [1]–[4]. Since mortality in ARDS patients remains high (30–45%) [3]–[5] further studies on the mechanisms that couple mechanical ventilation and inflammation are required in order to devise novel treatment strategies and to improve patient outcomes. The model of the isolated and perfused mouse lung (IPL) represents a common and widely used experimental tool to elucidate the mechanotransduction mechanisms related to VILI [6]–[9].

The IPL model permits to analyze the consequences of mechanical ventilation restricted to the lung tissue and results are not confounded by infiltrating blood cells or neuronal responses. For instance, in intact animals increased pulmonary MAPK kinase phosphorylation or gene expression may simply reflect the number of additional leukocytes that entered the lungs. A common disadvantage of perfused lung and *in vivo* experiments is the difficulty to obtain sequential samples from the lung tissue. Therefore, here we introduce the one-lung perfusion technique that allows to remove one lung for analysis while the other lung is continued to be perfused and ventilated.

Inflammatory responses in mice do clearly depend on the mouse strain. Important differences between C57BL/6 and BALB/c relate to airway hyperresponsiveness [10], and responses to bacteria [11]–[13], viruses [14], [15] and parasites [16], [17]. Furthermore, BALB/c mice carry the H-2d locus while C57BL/6 mice carry the H-2b locus similar to differences among humans [18], [19]. Recently, Wolthuis *et al*. studied the effects of mechanical ventilation and different treatments in C57BL/6 and BALB/c mice, but did not directly compare the inflammatory responses between these two strains [20]. Because of the “bias” in the immune system of these two mouse strains, the comparison of their responses to mechanical ventilation may provide further insight into the mechanisms of biotrauma.

One particularly relevant mechanotransduction mechanism during VILI is the activation of MAPK enzymes and subsequent pro-inflammatory gene transcription [1], [21], [22] However, many of these studies were performed *in vivo* and hence increased MAPK activation and protection by their inhibition might be explained on the level of leukocytes that require MAPK enzymes for their sequestration, migration and activation [23], [24] rather than by responses of the lung tissue itself.

The present study was designed to further define the role of MAPK enzymes and pulmonary mediator production in response to overventilation in two commonly used mouse strains. The mediators that we measured were selected because they are known to be either highly upregulated during mechanical ventilation, e.g Il6, Cxcl2, Cxcl1, amphiregulin, and/or because of their role in biotrauma, e.g. Il1b and Tnf [6], [25], [26]. Because MAPK activation precedes gene induction and cytokine production, here we used the one-lung technique to measure first MAPK activation in the left lung and 60 min later gene induction in the right lung from the same animal. These studies were performed in lungs from both C57BL/6 and in BALB/c mice that were subjected to either low or high tidal volume ventilation. Because backward regression analysis of these experiments suggested that p38 affects predominantly gene transcription rather than protein translation, we performed inhibitor experiments to further explore this hypothesis.

## Methods

### Animals

Female BALB/c and C57BL/6 mice weighting 20–25 g were obtained from Charles River (Charles River; Sulzfeld, Deutschland) and kept under standard conditions. All animal experiments were approved by the regional committee of animal experimentation ethics (LANUV, Landesamt für Umwelt, Natur und Verbraucherschutz, Recklinghausen, Nordrhein-Westfalen, Germany; Approval number 10509A6).

### Mechanical Ventilation and Perfusion

**Figure 1:**
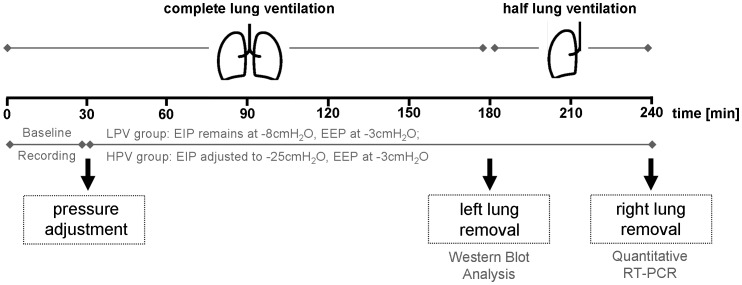
Study design – sample taking and processing. C57BL/6 and BALB/c mice were ventilated with negative pressure for a total of 240 minutes in the isolated perfused mouse lung setup. After 180 minutes the chamber was opened and a ligature was set around the left main bronchus, pulmonary artery and veins. The left lung was removed, powdered in liquid nitrogen and used for Western Blot analysis. The chamber was closed again and the right lung was ventilated for another 60 minutes. By the end of experiment the right lung was removed and powdered, RNA was extracted and qRT-PCR performed. During the entire experiment the lungs were perfused with HES-buffer with constant flow.

Ventilation experiments were performed using an isolated perfused mouse lung system (IL-1, Hugo Sachs Elektronik, March-Hugstetten, Germany). The preparation of mouse lungs was performed as described [27], [9]. Lungs were perfused in a non-circulating manner with constant flow of 1 mL per minute with custom made HES-buffer (SERAG-Wiessner, Naila, Germany). In all groups baseline ventilation was performed using negative pressure ventilation with an end-inspiratory pressure (EIP) of −8 cmH_2_O and an end-expiratory pressure (EEP) of −3 cmH_2_O resulting in a tidal volume of approximately 200 µL, and deep breath (30 cm H_2_O) every five minutes to prevent atelectasis [28]. The breathing frequency was 90 min^−1^ with an inspiration time of 50% of every breathing cycle. After 30 minutes the ventilation was randomly adjusted for the next 210 minutes to the following ventilation protocols (Fig. 1).

In control groups low pressure ventilation, as used in the baseline recordings, was continued (Low Pressure Ventilation  =  LPV). In the high pressure group, the ventilation parameters were adjusted to an end-inspiratory pressure of −25 cmH_2_O and an end-expiratory pressure of −3 cmH_2_O (High Pressure Ventilation  =  HPV). During left lung removal after 180 minutes ventilation was switched to positive pressure ventilation. The chamber was opened and a ligature placed around the left main bronchus, the left pulmonary artery, the left pulmonary veins before the left lung was removed, snap-frozen in liquid nitrogen immediately and stored at −80°C. The chamber was closed again and ventilation continued with the appropriate negative pressures. During the entire experiment lung perfusate samples were taken from a venous catheter every 30 minutes and stored at −20°C until further analyzed. After completion of the total ventilation time of 240 minutes bronchoalveolar lavage fluid (BALF, stored at −20°C) was obtained from the right lung by lavage with 500 µL phosphate buffered saline (PBS), before the lung was snap-frozen in liquid nitrogen and stored −80°C.

In a second series of experiments, BALB/c mice were ventilated with high pressure and the lungs were perfused with 0.5 mM p38 MAPK inhibitor SB203580 (Tocris Bioscience, Bristol, United Kingdom) dissolved in 1% DMSO in sterile saline or with solvent alone (control). Sample withdrawal and processing were carried out as described above.

### Enzyme-Linked Immunosorbent Assays (ELISA)

For detection of chemokine ligand-1 (CXCL1, KC), chemokine (C-X-C motif) ligand-2 (CXCL2, MIP-2), interleukin 6 (IL6), interleukin 1 beta (IL1b), tumor necrosis factor (TNF) and amphiregulin (AREG) in perfusate and BAL samples, commercially available ELISA kits were purchased (R&D Systems GmbH, Wiesbaden-Nordenstadt, Germany) and performed according to the manufacturers protocol.

### Immunoblot Analysis

Kinase activity was measured by Western blot analysis. Left lung samples were homogenized in liquid nitrogen and 20 mg of the resulting lung powder was used to extract proteins using cell extraction buffer (Invitrogen GmbH, Karlsruhe, Germany). Protein concentrations were measured using a bicinchoninic acid (BCA) kit (Interchim SA, Montluçon, France). For electrophoretic separation, 20 µg protein was supplemented with the denaturing and reducing loading buffer Roti®-Load 1 (Carl Roth GmbH & Co. KG, Karlsruhe, Germany) and subjected to electrophoresis in a 12% SDS gel. Separated proteins were transferred to a nitrocellulose membrane in a semi-dry method. Membranes were incubated overnight with monoclonal mouse antibodies against phospho-p44/42 MAPK (ERK1/2), phospho-Akt, phospho-p38 MAPK, phospho-SAPK/JNK. All mouse primary antibodies (Cell Signaling Technology, Inc., Danvers, USA) were diluted according to manufacturer recommendations. Horse radish peroxidase (HRP)-conjugated anti-mouse antibodies (Rockland, Gilbertsville, PA, USA) were used according to manufacturer’s recommendations as secondary antibodies for the detection of the phosphorylated kinases. After chemiluminescence detection by the use of a LAS-3000 Imager, membranes were stripped and incubated with monoclonal antibodies against the non-phosphorylated kinases pan-Akt, SAPK/JNK and polyclonal mouse antibodies against p44/42 MAPK (ERK1/2) and p38 MAP kinase. HRP-linked anti-rabbit antibodies (Rockland, Gilbertsville, PA, USA) were used for the detection of non-phosphorylated kinases using the LAS-3000 Imager. Pictures were evaluated with AIDA-Imager Software 4.0. Data were expressed as the ratio of the phosphorylated and the native form of the respective kinase; these data were then normalized to the BALB/c HPV group.

### Quantitative RT-PCR

Right lung samples were homogenized in liquid nitrogen and 20 mg of lung powder was prepared for automated RNA extraction and transferred to a QiaShredder spin column and automated RNA extraction was performed using the QIAcube (QIAGEN GmbH, Hilden, Germany) equipped with a RNeasy Mini-kit (Machery Nagel GmbH & Co. KG, Düren, Germany) using oligo(dT)_15_ Primer (Promega GmbH, Mannheim, Germany) according to manufacturers recommendations. RNA concentration was verified using the NanoDrop 1000 Spectrophotometer (Thermo Fisher Scientific Inc., Waltham, MA, USA). Real-time qPCR was performed in a LightCycler 480 (Roche-Diagnostics GmbH) according to the manufactureŕs instructions using the DNA-binding dye SYBR Green. The PCR reaction was performed in a total volume of 10 µL containing 5.0 µL 2x SYBR-Green Mastermix I (Roche-Diagnostics GmbH, Mannheim, Germany), 3.0 µL water, 0.5 µL customized-synthesized forward primer (6.25 µM) and 0.5 µL of the corresponding reverse primer (6.25 µM) and 1 µL cDNA template (equates to 50 ng cDNA). Primer sequences are listed in table 1. Gene expression was calculated according to the efficiency corrected equation published by Pfaffl. *et al* [29]. PCR efficiency was over 90% in all cases in all cases and determined by the standard curve method. The data were first normalized to the reference gene *rps29,* which was in house validated as the most stable reference gene in previous studies in our IPL setup and gene expression is displayed as fold induction in relation to control ventilated (NV group) Balb/C mice.

**Table 1:** Primer sequences used in this study.

	Sense	Antisense
***Rsp29***	5′-CCTTTCTCCTCGTTGGGCG-3′	5′-GAGCAGACGCGGCAAGAG-3′
***Cxcl1***	5′-CAGACAGTGGCAGGGATTC-3′	5′-TTCAGGGTCAAGGCAAGC-3′
***Cxcl2***	5′-AATGCCTGACGACCCTACCA-3′	5′GTTAGCCTTGCCTTTGTTCAG-3′
***Il6***	5′-CCAGCCAGTTGCCTTCTTG-3′	5′-AGTGCATCATCGCTGTTCATAC-3′
***Tnf***	5′-GGGGCCACCACGCTCTTCTGTCTA-3′	5′-CCTCCGCTTGGTGGTTTGCTACG-3′
***Areg***	5′-CTATCTTTGTCTCTGCCATCA-3′	5′-AGCCTCCTTCTTTCTTCTGTT-3′
***Il1b***	5′-GAAAGCTCTCCACCTCAATG-3′	5′-GCCGTCTTTCATTACACAGG-3′

### Statistical Analysis

Data analyses were performed using JMP 9.03 (SAS Institute, Cary, NC, USA). Data are given as mean + SEM. The first batch of experiments were performed with n = 6 per study group. Homoscedasticity was checked by the Bartlett test and if necessary a Box-Cox transformation was performed to allow parametric two-way ANOVA analysis (factors: strain, ventilation). If a significant interaction (strain × ventilation) was found, treatment effects were compared using Student’s t-test on LS Means Differences and p-values were adjusted by the fdr-procedure. For tidal volume the area under the curve (AUC) was used for univariate analyses.

In order to exploit the one-lung approach and to correlate the kinase activation data with gene expression and with protein production, we performed a backward regression analysis in which we used the kinase phosphorylation data (ERK, JNK, p38, Akt) as independent variables and either the PCR or the ELISA data as dependent variables. The best model was selected according to the BIC criterion. Backward regression is preferred over forward regression when studying modest group numbers; it may select a set of variables even when single variables have no predictive capability. Of note, backward regression is a data mining technology that needs independent confirmation. Here, backward regression analysis led to the hypothesis that p38 affects primarily gene expression that was tested with a group size of n = 5 per group. In all experiments p<0.05 was considered significant.

## Results

### Lung Functions

**Figure 2:**
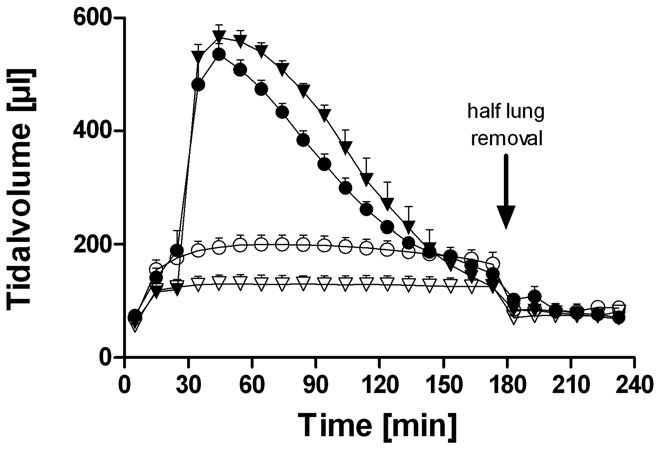
Tidal volume of C57BL/6 and BALB/c mice during low and high pressure ventilation. Data are represented as mean + SEM with n = 6 per study group. The differences in tidal volume and compliance between LPV and HPV were significant (p<0.01; two way ANOVA). BALB/c and C57BL/6 mice did not differ in tidal volume. C57BL/6 =  triangles; BALB/c  =  circles; LPV  =  open symbols; HPV  =  closed symbols.

Tidal volume remained stable over the entire ventilation period in the LPV group with no differences between the two strains (Fig. 2A). HPV increased the tidal volume, again with no difference between the two strains. As described before [27], [30], because we used pressure-control ventilation, tidal volumes decreased during overventilation. At the time of removal of the left lung, the tidal volumes had returned nearly back to baseline. After left lung removal, all of the remaining right lungs received similar tidal volumes of about 80 µL. Tidal volume and compliance did not differ in BALB/c mice when perfused with the p38 MAP kinase inhibitor SB203580 or the solvent DMSO (data not shown).

### Kinase Activity

**Figure 3:**
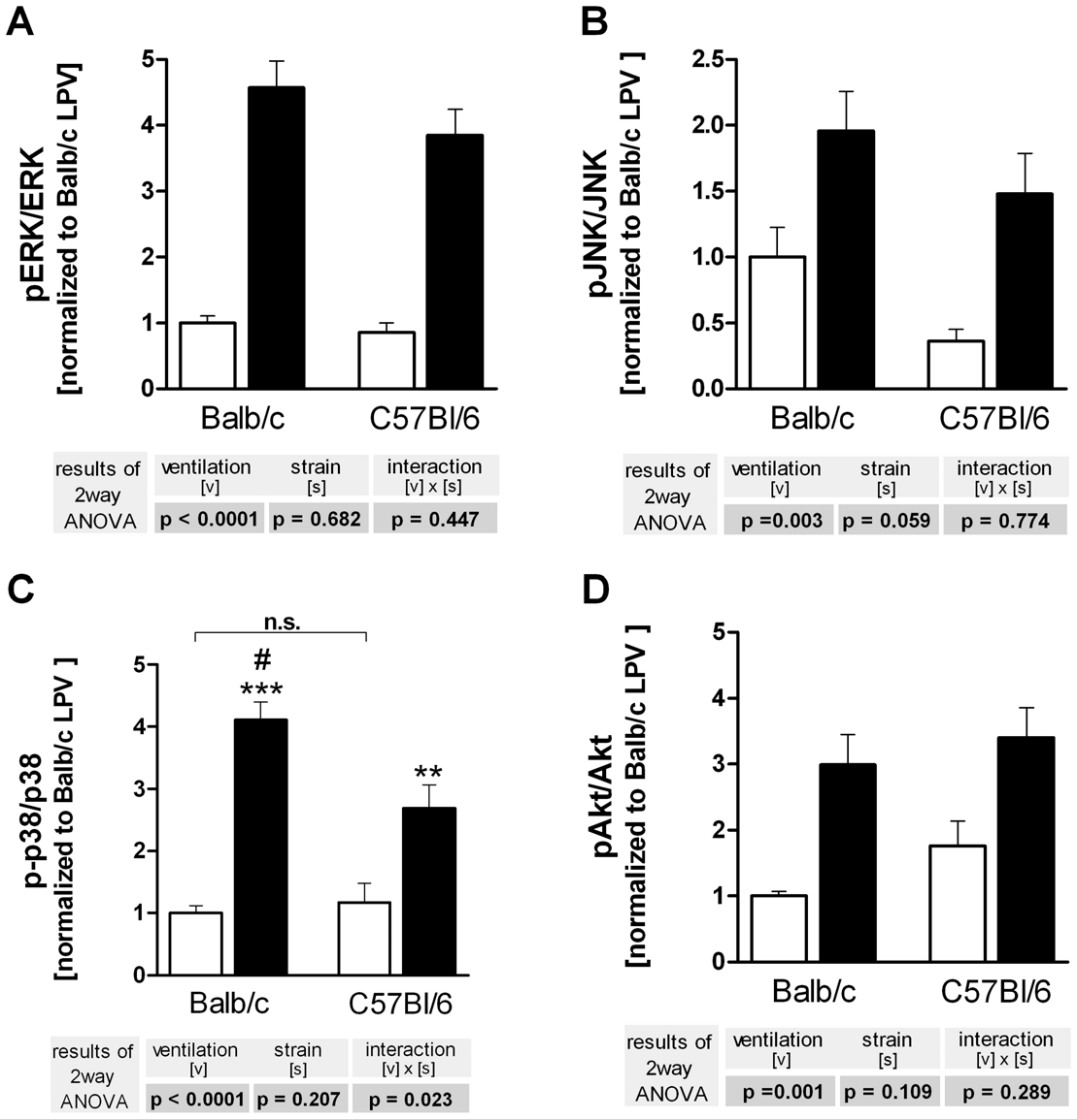
Ventilation and strain-dependent differences in kinase phosphorylation in C57BL/6 and BALB/c mice. Left lungs were harvested after 180 min of ventilation and perfusion and probed for phosphorylation of ERK (**A**), JNK (**B**), p38 (**C**) and Akt (**D**). LPV, white bars; HPV, black bars. Data are mean + SEM with n = 6 per study group. Data are expressed as the ratio of the phosphorylated and the native form of the respective kinase; these data were then normalized to the BALB/c HPV group. Two way ANOVA was performed, and only if a significant interaction (strain × ventilation) was detected, individual comparisons were made (*P<0.05; **P<0.01; ***P<0.001 vs. LPV; #*P*<0.01 vs. C57BL/6).

Phosphorylation of ERK, JNK, p38 and Akt was strongly increased in both strains during HPV (Fig. 3). Interstrain differences were observed for p38 that was stronger phosphorylated in BALB/c mice (Fig. 3C). Original blots are given in the supporting material (Fig. S1).

### Gene Expression

**Figure 4:**
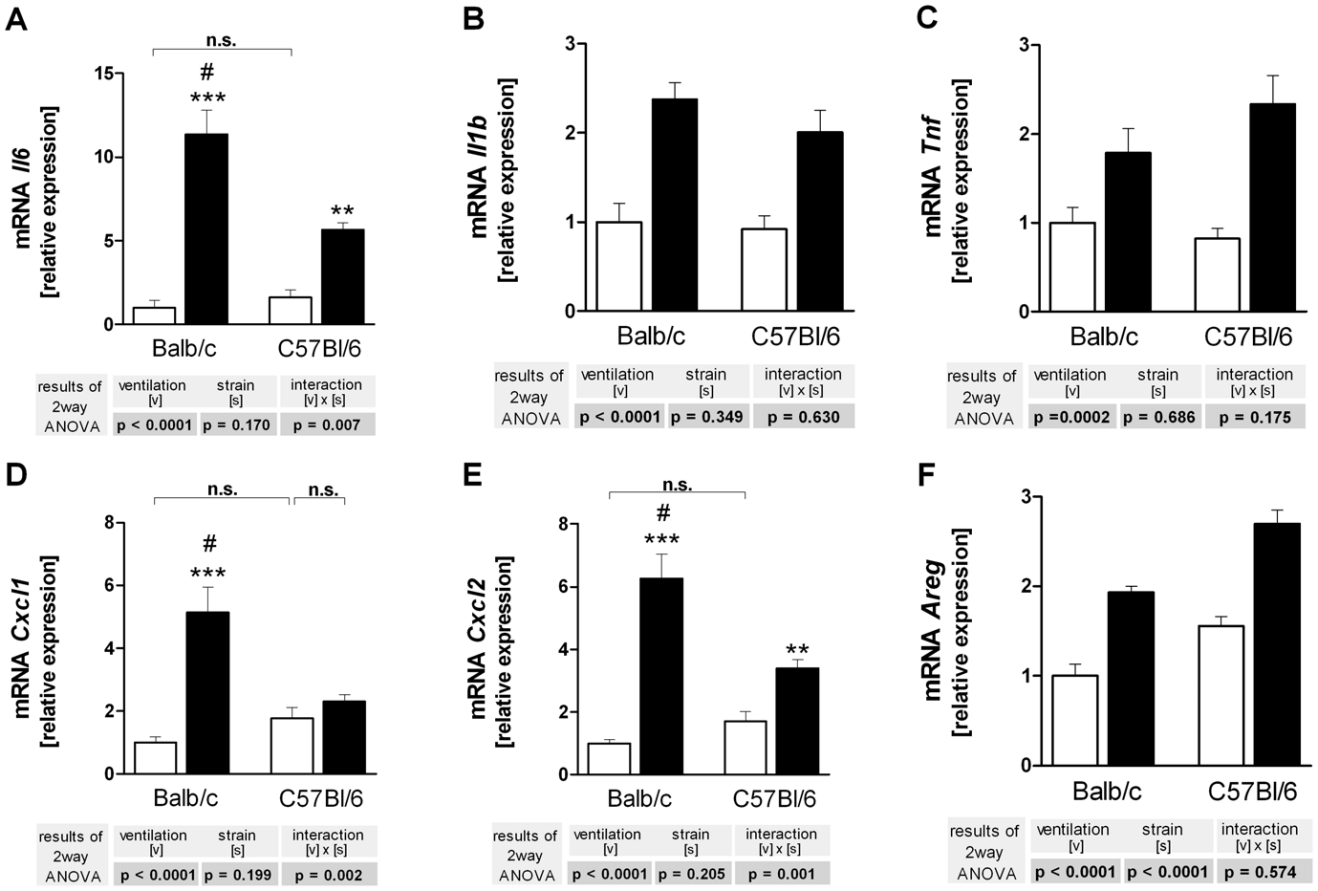
Gene expression of cytokines (A–C), chemokines (D,E) and amphiregulin (F) in C57BL/6 and BALB/c mice. Right lungs were harvested after 240 min of ventilation and perfusion and probed by RT-qPCR for *Il6* (**A**), *Il1b* (**B**), *Tnf* (**C**), *Cxcl1* (**D**), *Cxcl2* (**E**) and *Areg* (**E**). LPV, white bars; HPV, black bars. Data are mean + SEM with n = 6 per study group. Two way ANOVA was performed and only if a significant intercept (strain × ventilation) was detected, it was followed by individual comparisons (*P<0.05; **P<0.01; ***P<0.001 vs. LPV; #*P*<0.01 vs. C57BL/6).

Baseline gene expression was comparable in both mouse strains. HPV induced marked expression of all genes studied here, e.g. *Il6*, *Il1b, Tnf, Cxcl1, Cxcl2* and *Areg* (Fig. 4). Strain differences were noted for the overventilation-induced expression of *Il6, Cxcl1* and *Ccxl2* that was stronger in BALB/c mice.

### Cytokines in BAL

**Figure 5:**
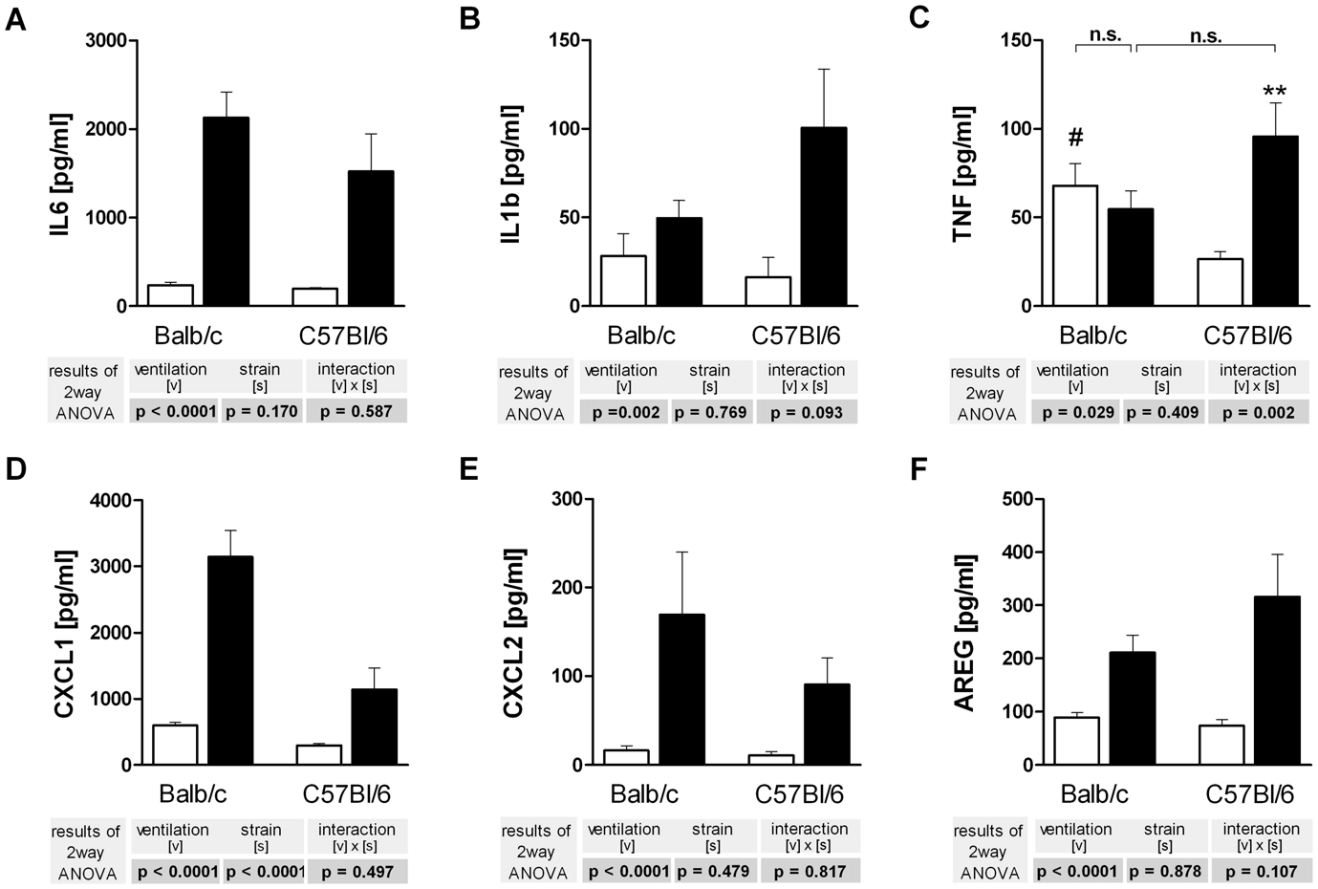
Cytokine (A–C), chemokine (D,E) and amphiregulin (F) concentrations in the bronchoalveolar fluid of C57BL/6 and BALB/c mice. Lung lavage fluid was obtained from the right lung after 240 min of ventilation and analyzed by ELISA for IL6 (**A**), Il1b (**B**), TNF (**C**), CXCL1 aka KC (**D**), CXCL2 aka MIP-2α (**E**) and amphiregulin (**E**). LPV, white bars; HPV, black bars. Data are mean + SEM with n = 6 per study group. Two way ANOVA was performed and only if a significant intercept (strain × ventilation) was detected, it was followed by individual comparisons (*P<0.05; **P<0.01; ***P<0.001 vs. LPV; #*P*<0.01 vs. C57BL/6).

Recovery of BAL fluid was approximately 80% in all animals with no differences between groups. Baseline protein levels in the BAL were similar for both mouse strains, except for TNF protein that was higher in BALB/c mice. Overventilation increased the BAL levels of IL6, IL1b, CXCL1 (KC), CXCL2 (MIP-2) and amphiregulin in both mouse strains. TNF protein in the BAL fluid was increased by overventilation only in C57BL/6 mice (Fig. 5).

### Backward Regression

A great advantage of the one-lung technique is that there are two tissue samples from the same animal taken at different time points. This offers the possibility to correlate in the same animal signaling events such as kinase activation to subsequent events such as gene expression and protein production. In order to exploit this opportunity we used the kinase data from figure 3 and calculated by backward regression which of the changes in kinase activation could possibly explain the changes seen in gene expression (Fig. 4) and protein production (Fig. 5).

This analysis (Table 1) showed that 64 to 81% of the changes in the expression of *Il6, Cxcl1, Cxcl2* and *Il1b* were explained by alterations in the phosphorylation status of p38, whereas protein translation was better explained by variations in ERK phosphorylation. These correlation data should be viewed as hypotheses that require experimental verification. The observation that activation of p38 explained such a large part of PCR data, but only a marginal part of the BAL measurements, suggests the hypothesis that in our model p38 controls predominantly gene expression, but not protein production. This hypothesis was tested by examining the effects of the p38 inhibitor SB203580 in overventilated BALB/c mice.

### P38 Inhibitor Studies

**Figure 6:**
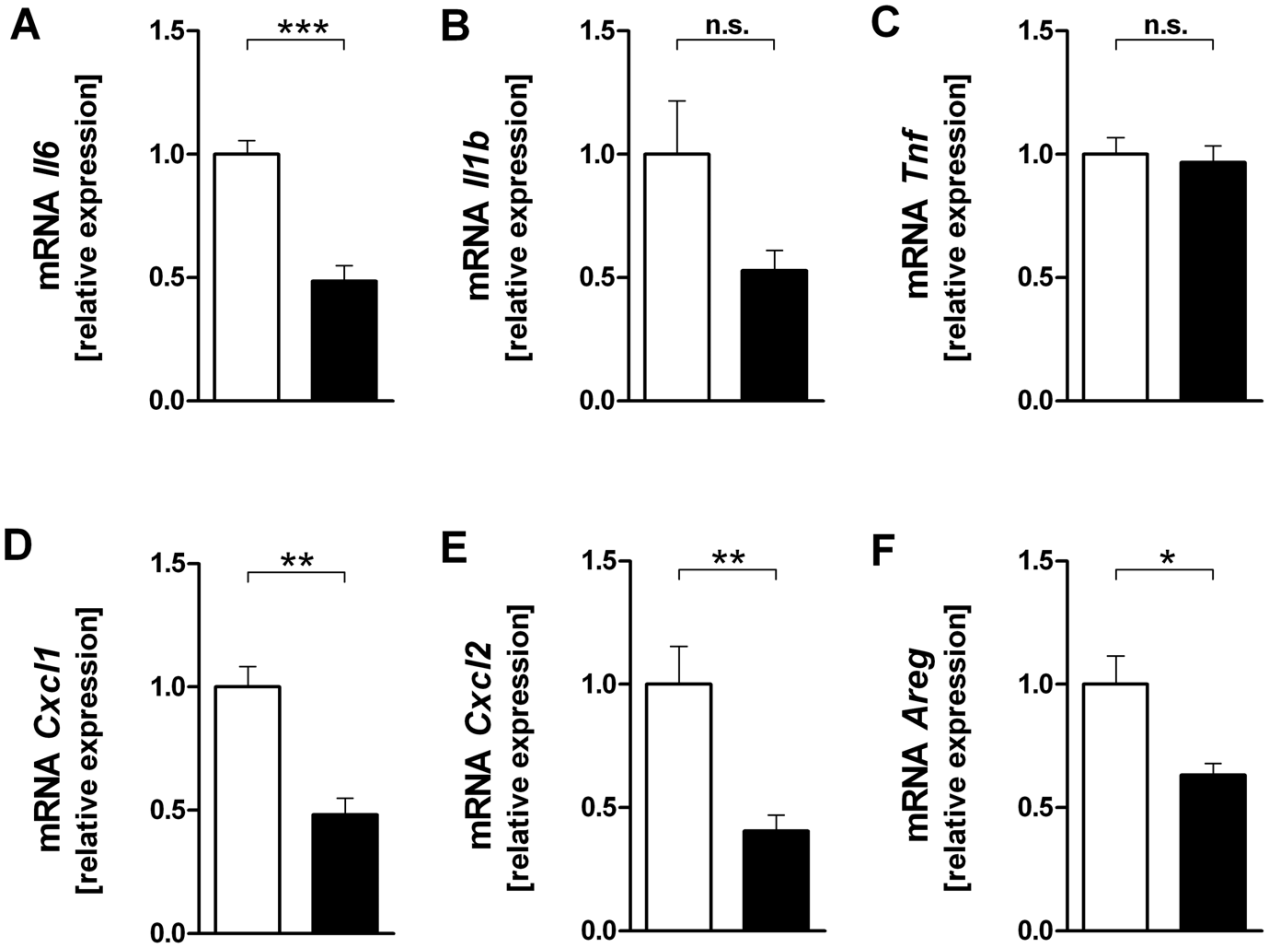
Effect of a p38 MAP kinase inhibitor on cytokine (A–C), chemokine (D,E) and amphiregulin (F) mRNA expression in high pressure ventilated BALB/c mice. All lungs were subjected to HPV. Right lungs were harvested after 240 min of ventilation and perfusion and probed by RT-qPCR for *Il6* (**A**), *Il1b* (**B**), *Tnf* (**C**), *Cxcl1* (**D**), *Cxcl2* (**E**) and *Areg* (**E**). LPV, vehicle (DMSO); HPV, SB203580. Data are mean + SEM with n = 6 per study group. (*P<0.05; **P<0.01; ***P<0.001).

**Figure 7:**
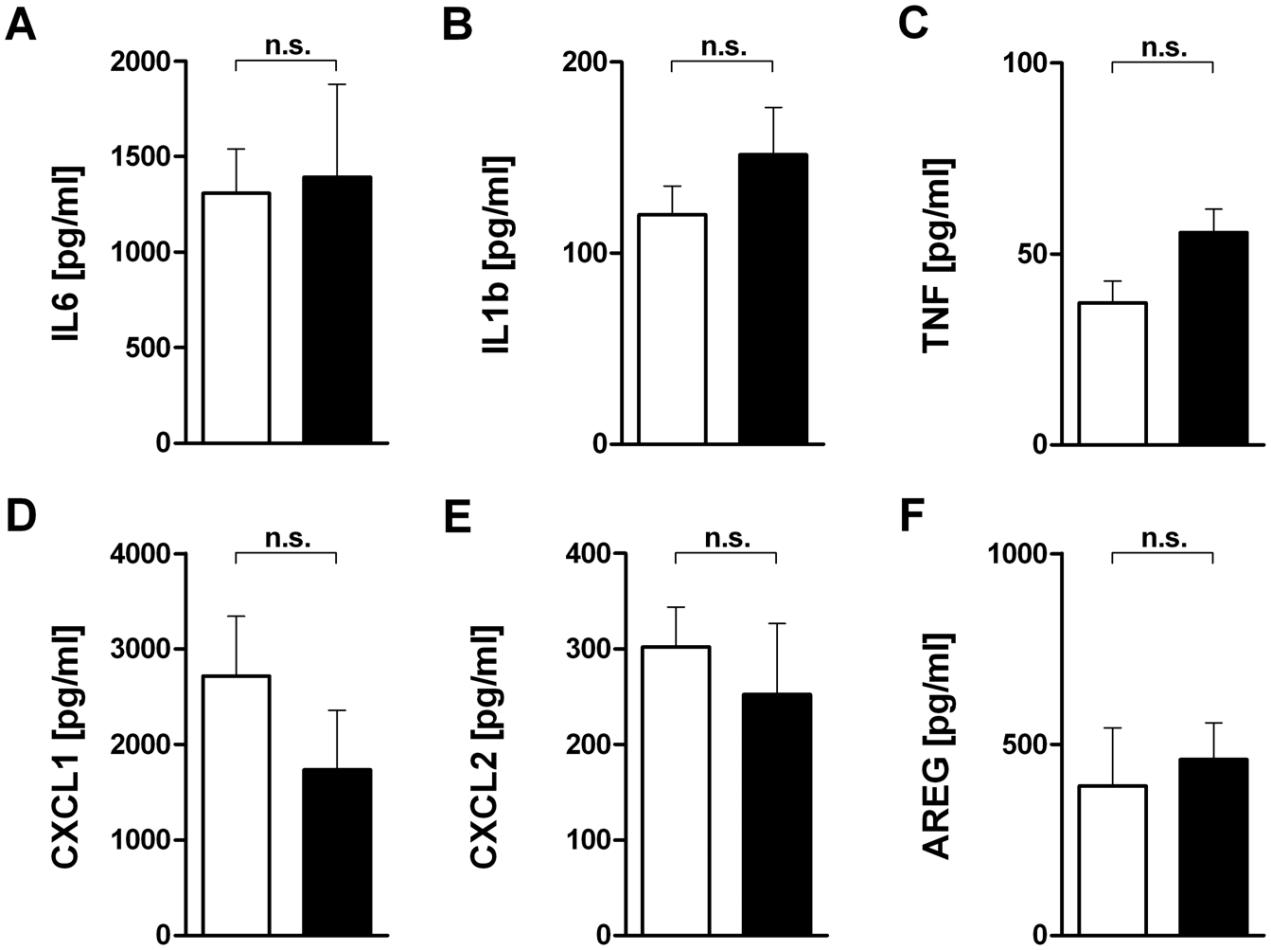
Effect of a p38 MAP kinase inhibitor on cytokine (A–C), chemokine (D,E) and amphiregulin (F) concentration in bronchoalveolar fluid in high pressure ventilated BALB/c mice. All lungs were subjected to HPV. Lung lavage fluid was obtained from the right lung after 240 min of ventilation and analyzed by ELISA for IL6 (**A**), Il1b (**B**), TNF (**C**), CXCL1 aka KC (**D**), CXCL2 aka MIP-2α (**E**) and amphiregulin (**E**). LPV, vehicle (DMSO); HPV, SB203580. Data are mean + SEM with n = 6 per study group.

Lungs of BALB/c mice were exposed to HPV and treated with SB203580 or DMSO. Treatment with SB203580 had no effect on the MAP kinase phosphorylation (data not shown), but significantly reduced the gene expression of *Il6, Cxcl1, Cxcl2* and *Areg* and showed a trend in case of *Il1b* (Fig. 6). SB203580 did however not significantly affect the protein concentrations in the BAL fluid (Fig. 7).

## Discussion

Here we have applied the novel method of one-lung perfusion and ventilation in mice to study differences between mouse strains in ventilator-induced signalling pathways. Our findings indicate that overventilation promotes pro-inflammatory gene expression and protein production in both strains and that for the greater part these responses are stronger in BALB/c than in C57BL/6 mice. Building on the possibility offered by the one-lung method to correlate kinase activation with genes and proteins in the same animal, we hypothesized that p38 does control mainly pro-inflammatory gene transcription, but not protein production. This hypothesis was confirmed in experiments with the p38-kinase inhibitor SB203580 indicating that during mechanical ventilation gene transcription and protein translation are differentially regulated by MAP kinases.

Isolated perfused mouse lungs have been established as a useful tool to examine the mechanisms of ventilator-induced lung injury. For instance, using this model it was shown that overventilation activates MAP kinases [21] and Akt kinase [31], [32]. Hitherto, however, kinase activation and the subsequent pulmonary gene and protein expression could not be studied in the same lungs, because they occur at different time points. This is a significant constraint, because it not only increases the demands in terms of animals and time, but it also rules out the possibility to employ the statistically so powerful paired analysis within the same animal. To overcome these limitations we have developed the one-lung perfusion technique. Our data show that stable ventilation and perfusion of one lung is feasible and that lung mechanics are not significantly altered if corrected for tidal volume. With this method it is now possible to obtain and analyse lung tissue from the same animal at two different time points.

In intact lungs low pressure ventilation resulted in a tidal volume of 200 µL in both strains. High pressure ventilation initially increased the tidal volume to 550 µL with a similar decline in both strains, a decline that might be partly explained by a loss of surfactant [33] and/or a loss of recruitable lung tissue. It is important to emphasize that this ventilation strategy does not cause gross lung injury or edema formation [27], [30]. The lack of substantial differences in lung mechanics between BALB/c and C57BL/6 mice is in line with previous studies in isolated lungs [34] and *in vivo* [35]. The fact that both mouse strains have comparable lung mechanics indicates that any differences between the strains in cytokine expression are probably due to differences in signalling rather than due to differences in the mechanical properties of their lungs.

It is well known that high tidal volume ventilation increases pro-inflammatory cytokine production in isolated lungs, in ventilated animals and in patients, a process that may lead to biotrauma, which is considered highly relevant to the pathogenesis of acute lung injury [1]. One important problem within this context relates to pro-inflammatory signalling cascades and whether they can be exploited therapeutically [36], [37]. Such studies have to rely heavily on animal models and –as shown here– the results may depend to some extent on the mouse strain used. First, our findings show that the activation of four important kinases (p38, JNK, ERK, Akt) was comparable between BALB/c and C57BL/6 mice, suggesting that the initial mechanotransduction pathways are similar in both mouse strains. Second, the induction of *Il6*, *Cxcl1 (KC)* and *Cxcl2 (MIP-2α)* genes by overventilation was stronger in BALB/c than in C57BL/6 mice (Fig. 3). The ventilation-induced increase of *Tnf* on the other hand, was slightly stronger in C57BL/6 mice. The discrepant regulation of different genes during overventilation has been shown before in studies with trichostatin A (an inhibitor of histone deacetylase) that enhanced the transcription of *Tnf* and *Cxcl2*, but diminished that of *Il6* [38], [39]; in addition, in a cluster analysis all three genes clustered separately [39]. Another example of the discrepant regulation of *Tnf* and *Il6* is given in ref. [9]. The mechanisms responsible for the divergent gene expression in response to exactly the same stimulus are poorly defined, and we speculate that acetylation of histones or other proteins [39] and kinases such as p38 (see below) might be involved.

Our finding that pro-inflammatory gene expression is stronger in BALB/c mice is in line with several previous studies that compared C67Bl/6 and BALB/c mice: After infection with *mcyoplasma pneumonia*, the BAL levels of CXCL1, MIP-1α, MCP-1, IL-1b and IL-6 were higher in BALB/c mice [40]. Similarly, Sendai virus-induced IL-6 and KC production was higher in mesangial cells from BALB/c mice [14]. In *Neisseria gonorrhoeae* infections, there were higher protein levels of IL-6, CXCL1 and CXCL2 in the vaginal secretions of BALB/c mice [41]. Conversely, similar to the present findings, macrophages from C57BL/6 mice produced more TNF after stimulation with LPS or MALP-2 (macrophage-activating lipopeptide-2; TLR-2 ligand) [42]. It should be noted, however, that in the *M. pneumonia* and the *Neisseria gonorrhoeae* studies TNF expression was higher in BALB/c mice [40], [41]. Thus, with the possible exception of TNF, BALB/c mice apparently show stronger cytokine and chemokine responses than do C57BL/6 mice, an observation that in some cases also translated into a stronger inflammatory response [40], [41], although this was not observed during ventilation of mice with 15 mL/kg V_T_ [20], which however must be considered a moderate stimulus [28]. A possible explanation for the increased cytokine production in BALB/c mice might be the fact that C57BL/6 mice lack the secretory phospholipase A_2_ [43] that contributes to inflammatory responses in acute lung injury [44].

The role of MAP kinases in models of acute lung injury has been examined in several *in vivo* studies before. In such studies inhibition of p38 MAP kinase attenuated lung injury and cytokine levels in various models such burn trauma [45], LPS treatment [46] and VILI (see below). Inhibition of p38 MAP kinase improved acute lung injury and reduced BAL inflammatory cytokine responses (as shown by reduced TNF-α, and IL-1β) levels in burn trauma induced lung injury [45] and inhibition of LPS-induced airway neutrophil infiltration and IL-6 recovery from BAL fluid in guinea pig [47] due to inhibition of the kinase. In a murine model of mild LPS-induced lung inflammation systemic inhibition of p38 MAPK resulted in significant decreases in TNF-α release but had no affect on CXCL1 and CXCL2 levels [48]. Unchanged or opposite effects on cytokine levels were shown in a murine model of pneumococcal pneumonia and tuberculosis in which increased TNF-α levels were observed after p38 MAPK inhibition but this effect was absent in LPS treated mice [49]. p38 seems not to have a central role in the development of ALI as shown by unchanged proinflammatory cytokine levels despite p38 inhibition after hemorrhage or endotoxemia [50]. These studies, which were all performed in C57BL/6 mice, have shown that treatment with SB203580 decreased vascular permeability, PARP-1 cleavage and apoptosis [51], [52] during injurious ventilation, but mediators were not measured. In all these studies it is unclear whether SB203580 acted on the lungs tissue or on the invading neutrophils. The latter possibility is underscored by studies in mice deficient in MKK3^−/−^ (which is upstream of p38, although other mechanisms may activate p38 MAPK as well [53]) that showed less sequestration of PMNs, less apoptosis and less TNF in their lungs, but the same degree of pulmonary edema [54]. Similarly, JNK1^−/−^ mice did also show diminished BAL neutrophils together with diminished expression of MIP-2 [55]. Thus, these previous studies showed that p38 and JNK do contribute to leukocyte infiltration in VILI, but they did not clarify the relative roles of neutrophilic versus pulmonary MAP kinases.

The one-lung set-up provided the opportunity to approach this question, because it allows to correlate, for instance by regression analysis, signalling events at one time point with gene expression and mediator production at a later time point in the same animal and in the absence of blood-borne leukocytes. Backward regression of gene expression data against the kinase data showed that variations in p38 explained approximately more than two thirds of the variations in the transcription of *Il1b, Il6, Cxcl1 and Cxcl2*, suggesting that during overventilation these genes are regulated by p38. Since the corresponding proteins in the BAL did not correlate with p38 (table 2), we predicted that SB203580 would affect gene transcription, but not protein production. This hypothesis was entirely confirmed by the experiments shown in figures 6 and 7. These data show a dissociation between gene expression and protein production, and indicate that to some extent gene expression is regulated by p38, whereas translation is regulated by other factors, e.g. by Erk. A technical explanation for the dissociation between transcription and translation would be to assume that BAL protein levels do not reflect tissue protein levels. While we cannot completely exclude this possibility, Wolthuis *et al*. following ventilation with 15 mL/kg, showed that tissue and BAL cytokine levels (TNF, IL6, CXCL1, CXCL2) were similar in both Blab/C and C57BL/6 mice [20]. In addition, we observed in another study a situation where *Tnf* and *Cxcl2* gene expression was increased, but protein release into the perfusate was unaltered or even decreased [39].

**Table 2:** Percent of the variations in gene and protein expression that are explained by variations in kinase phosphorylation.

		p38		JNK		ERK		Akt		Unexplained
**Il6**	gene	81%	***	4%	*	–		–		15%
	protein	3%		–		81%	**	–		16%
**Cxcl1**	gene	67%	***	–		–		–		33%
	protein	–		5%	*	69%	***	5%	*	21%
**Cxcl2**	gene	75%	***	–		–		6%	*	19%
	protein	–		–		53%	***	–		47%
**Il1b**	gene	64%	***	9%		–		4%		23%
	protein	–		–		30%	**	–		70%
**Tnf**	gene	–		–		41%	***	–		69%
	protein	–		27%	**	–		–		73%
**Areg**	gene	–		7%		48%	***	–		45%
	protein	–		–		58%	***	–		42%

Gene refers to PCR data and protein to the ELISA measurements in the BAL fluid. Backward regression was performed for each of the genes and each of the proteins separately, in each case with all four kinases as the independent variables. The percent of the variations explained correspond to the squared semipartial correlation coefficients (*P<0.05; **P<0.01; ***P<0.001).

All these data favour the hypothesis that gene transcription and protein production in the lungs are regulated separately. There at least three different possibilities to explain such a dissociation between translation and transcription: (i) Proteins may exist as a preform or can be stored as was suggested for CXCL2 and IL1β [56]. (ii) It is known that p38 may stabilize mRNA via phosphorylation of tristetraprolin [57]. In this scenario, a p38 inhibitor such as SB203580 need not affect transcription and hence sufficient mRNA might be available for translation at some time point; the mRNA, however, is then rapidly degraded until the time when measured. (iii). According to our analysis, 15–33% of gene transcription was independent of p38; in the experiments with by SB203580 gene expression was halved as compared to a several fold increase by overventilation as shown in fig. 4. Thus, inhibition of gene transcription was most likely not complete and the available mRNA might be sufficient to fuel translation.

Our findings that p38 inhibitor application did not impact on pro-inflammatory cytokine responses are in line with several previous studies in LPS-induced lung injury, where inhibition of p38 did affect neither the BAL levels of MIP-2 or KC nor neutrophil sequestration into the lungs [50], [58], although it reduced the number of neutrophils in the lavage [58]. These findings indicate that beneficial effects of p38 inhibition in lung injury models [45], [46], [48] are possibly to a significant degree explained by the role of p38 in the chemotaxis of neutrophils [58] and less by effects on resident lung cells. However, there are some cytokines, in particular TNF and IL-6, which have consistently been found to be decreased by p38-inhibition in models of ALI [46], [48]. The present findings indicate that these findings may be explained by the role of p38 in the invaded leukocytes that do either produce these cytokines themselves or interact with other cells to stimulate their production.

### Conclusions

One-lung perfusion is a useful and animal-saving method to study lung tissue from the same lung at two different time points. Using this novel method we show that while lung mechanics and mechanotransduction are similar in BALB/c and C57BL/6 mice, the production of pro-inflammatory mediators is stronger in BALB/c mice. The hypo-inflammatory phenotype of C57BL/6 mice needs to be taken into account when performing and interpreting studies on ventilator-induced lung injury in this strain. We further show that p38 controls the overventilation-induced expression of four pro-inflammatory genes, namely *Il1b, Il6, Cxcl1* and *Cxcl2*. The dissociation between gene expression and protein production illustrates a complex regulatory network and demonstrates that gene expression data alone need to be interpreted with caution.

## Supporting Information

Figure S1 **Ventilation and strain-dependent differences in kinase phosphorylation in C57BL/6 and BALB/c mice.** Left lungs were harvested after 180 min of ventilation and perfusion and probed for the native and the phosphorylated form of ERK (**A**), JNK (**B**), p38 (**C**) and Akt (**D**). C57BL/6 =  triangles; BALB/c  =  circles; LPV  =  open symbols; HPV  =  closed symbols
(TIF)

## References

[1] Uhlig U, Uhlig S, Terjung R (2011). Ventilation-Induced Lung Injury.. http://www.comprehensivephysiology.com/WileyCDA/CompPhysArticle/refId-c100004.html.

[2] The Acute Respiratory Distress Syndrome Network (2000). Ventilation with Lower Tidal Volumes as Compared with Traditional Tidal Volumes for Acute Lung Injury and the Acute Respiratory Distress Syndrome.. N Engl J Med.

[3] Villar J, Blanco J, Añón JM, Santos-Bouza A, Blanch L (2011). The ALIEN study: incidence and outcome of acute respiratory distress syndrome in the era of lung protective ventilation.. Intensive Care Med.

[4] Pierrakos C, Vincent J-L (2012). The changing pattern of ARDS over time: A comparison of two periods. The European Respiratory Journal: Official Journal of the European Society for Clinical Respiratory Physiology.. http://www.ncbi.nlm.nih.gov/pubmed/22323569.

[5] Donahoe M (2011). Acute respiratory distress syndrome: A clinical review.. Pulm Circ.

[6] Dolinay T, Kaminski N, Felgendreher M, Kim HP, Reynolds P (2006). Gene expression profiling of target genes in ventilator-induced lung injury.. Physiol Genomics.

[7] Fanelli V, Puntorieri V, Assenzio B, Martin EL, Elia V (2010). Pulmonary-derived phosphoinositide 3-kinase gamma (PI3Kγ) contributes to ventilator-induced lung injury and edema.. Intensive Care Med.

[8] Rocca NA, Walker MG, McCaig LA, Yao L-J, Potter RF (2011). The biological effects of lung-derived mediators on the liver.. Exp Lung Res.

[9] Von Bethmann, AN Brasch, F Müller, K Wendel, A Uhlig, S (1996). Prolonged hyperventilation is required for release of tumor necrosis factor a but not IL-6.. Appl Cardiopulm Pathol.

[10] Säfholm J, Lövdahl C, Swedin L, Boels PJM, Dahlén S-E (2011). Inflammation-induced airway smooth muscle responsiveness is strain dependent in mice.. Pulm Pharmacol Ther.

[11] Wakeham J, Wang J, Xing Z (2000). Genetically Determined Disparate Innate and Adaptive Cell-Mediated Immune Responses to Pulmonary Mycobacterium Bovis BCG Infection in C57BL/6 and BALB/C Mice.. Infect Immun.

[12] Bohn E, Heesemann J, Ehlers S, Autenrieth IB (1994). Early Gamma Interferon mRNA Expression Is Associated with Resistance of Mice Against Yersinia Enterocolitica.. Infect Immun.

[13] Van Doorn NEM, Namavar F, Sparrius M, Stoof J, Van Rees EP (1999). Helicobacter Pylori-Associated Gastritis in Mice Is Host and Strain Specific.. Infect Immun.

[14] Kobayashi N, Bagheri N, Nedrud JG, Strieter RM, Tomino Y (2003). Differential effects of Sendai virus infection on mediator synthesis by mesangial cells from two mouse strains.. Kidney International.

[15] Weinberg JB, Lutzke ML, Alfinito R, Rochford R (2004). Mouse strain differences in the chemokine response to acute lung infection with a murine gammaherpesvirus.. Viral Immunol.

[16] Roggero E, Perez A, Tamae-Kakazu M, Piazzon I, Nepomnaschy I (2002). Differential susceptibility to acute Trypanosoma cruzi infection in BALB/c and C57BL/6 mice is not associated with a distinct parasite load but cytokine abnormalities.. Clin Exp Immunol.

[17] Rossi-Bergmann B, Müller I, Godinho EB (1993). TH1 and TH2 T-cell subsets are differentially activated by macrophages and B cells in murine leishmaniasis.. Infect Immun.

[18] McDevitt HO, Chinitz A (1969). Genetic Control of the Antibody Response: Relationship Between Immune Response and Histocompatibility (H-2) Type.. Science.

[19] Hill AVS (1998). The Immunogenetics of Human Infectious Diseases.. Annual Review of Immunology.

[20] Wolthuis EK, Vlaar APJ, Choi G, Roelofs JJTH, Juffermans NP (2009). Mechanical ventilation using non-injurious ventilation settings causes lung injury in the absence of pre-existing lung injury in healthy mice.. Crit Care.

[21] Uhlig U, Haitsma JJ, Goldmann T, Poelma DL, Lachmann B (2002). Ventilation-induced activation of the mitogen-activated protein kinase pathway.. European Respiratory Journal.

[22] Abdulnour R-EE, Peng X, Finigan JH, Han EJ, Hasan EJ (2006). Mechanical stress activates xanthine oxidoreductase through MAP kinase-dependent pathways.. American Journal of Physiology - Lung Cellular and Molecular Physiology.

[23] Herlaar E, Brown Z (1999). p38 MAPK signalling cascades in inflammatory disease.. Mol Med Today.

[24] Yong H-Y, Koh M-S, Moon A (2009). The p38 MAPK inhibitors for the treatment of inflammatory diseases and cancer.. Expert Opinion on Investigational Drugs.

[25] Wilson MR, Goddard ME, O’Dea KP, Choudhury S, Takata M (2007). Differential roles of p55 and p75 tumor necrosis factor receptors on stretch-induced pulmonary edema in mice.. Am J Physiol Lung Cell Mol Physiol.

[26] Frank JA, Pittet J-F, Wray C, Matthay MA (2008). Protection from experimental ventilator-induced acute lung injury by IL-1 receptor blockade.. Thorax.

[27] von Bethmann A, Brasch F, Nusing R, Vogt K, Volk H (1998). Hyperventilation Induces Release of Cytokines from Perfused Mouse Lung.. Am J Respir Crit Care Med.

[28] Reiss LK, Kowallik A, Uhlig S (2011). Recurrent Recruitment Manoeuvres Improve Lung Mechanics and Minimize Lung Injury during Mechanical Ventilation of Healthy Mice.. PLoS ONE.

[29] Pfaffl MW (2001). A new mathematical model for relative quantification in real-time RT-PCR.. Nucleic Acids Res.

[30] Uhlig U, Drömann D, Goldmann T, Dombrowsky H, Vollmer E (2006). Pulmonary responses to overventilation in late multiple organ failure.. Anesthesiology.

[31] Kuebler WM, Uhlig U, Goldmann T, Schael G, Kerem A (2003). Stretch activates nitric oxide production in pulmonary vascular endothelial cells in situ.. Am J Respir Crit Care Med.

[32] Miyahara T, Hamanaka K, Weber DS, Drake DA, Anghelescu M (2007). Phosphoinositide 3-kinase, Src, and Akt modulate acute ventilation-induced vascular permeability increases in mouse lungs.. Am J Physiol Lung Cell Mol Physiol.

[33] Stamme C, Brasch F, von Bethmann A, Uhlig S (2002). Effect of surfactant on ventilation-induced mediator release in isolated perfused mouse lungs.. Pulm Pharmacol Ther.

[34] Held H-D, Uhlig S (2000). Basal lung mechanics and airway and pulmonary vascular responsiveness in different inbred mouse strains.. J Appl Physiol.

[35] Reinhard C, Eder G, Fuchs H, Ziesenis A, Heyder J (2002). Inbred strain variation in lung function.. Mamm Genome.

[36] Uhlig S, Uhlig U (2004). Pharmacological interventions in ventilator-induced lung injury.. Trends in Pharmacological Sciences.

[37] Reiss LK, Uhlig U, Uhlig S (n.d.) Models and mechanisms of acute lung injury caused by direct insults. European Journal of Cell Biology.. http://www.sciencedirect.com/science/article/pii/S0171933511002226.

[38] Dombrowsky H, Uhlig S (2007). Steroids and histone deacetylase in ventilation-induced gene transcription.. Eur Respir J.

[39] Dombrowsky H, Barrenschee M, Kunze M, Uhlig S (2009). Conserved responses to trichostatin A in rodent lungs exposed to endotoxin or stretch.. Pulm Pharmacol Ther.

[40] Fonseca-Aten M, Ríos AM, Mejías A, Chávez-Bueno S, Katz K (2005). Mycoplasma Pneumoniae Induces Host-Dependent Pulmonary Inflammation and Airway Obstruction in Mice.. Am J Respir Cell Mol Biol.

[41] Packiam M, Veit SJ, Anderson DJ, Ingalls RR, Jerse AE (2010). Mouse Strain-Dependent Differences in Susceptibility to Neisseria Gonorrhoeae Infection and Induction of Innate Immune Responses.. Infect Immun.

[42] Watanabe H, Numata K, Ito T, Takagi K, Matsukawa A (2004). Innate immune response in Th1- and Th2-dominant mouse strains.. Shock.

[43] Kennedy BP, Payette P, Mudgett J, Vadas P, Pruzanski W (1995). A natural disruption of the secretory group II phospholipase A2 gene in inbred mouse strains.. J Biol Chem.

[44] Kitsiouli E, Nakos G, Lekka ME (2009). Phospholipase A2 subclasses in acute respiratory distress syndrome.. Biochim Biophys Acta.

[45] Chen X-L, Xia Z-F, Ben D-F, Wang G-Q, Wei D (2003). Role of p38 mitogen-activated protein kinase in lung injury after burn trauma.. Shock.

[46] Liu S, Feng G, Wang G-L, Liu G-J (2008). p38MAPK inhibition attenuates LPS-induced acute lung injury involvement of NF-kappaB pathway.. Eur J Pharmacol.

[47] Underwood DC, Osborn RR, Bochnowicz S, Webb EF, Rieman DJ (2000). SB 239063, a P38 MAPK Inhibitor, Reduces Neutrophilia, Inflammatory Cytokines, MMP-9, and Fibrosis in Lung.. Am J Physiol Lung Cell Mol Physiol.

[48] Nick JA, Young SK, Brown KK, Avdi NJ, Arndt PG (2000). Role of P38 Mitogen-Activated Protein Kinase in a Murine Model of Pulmonary Inflammation.. J Immunol.

[49] Lee JC, Kassis S, Kumar S, Badger A, Adams JL (1999). p38 Mitogen-Activated Protein Kinase Inhibitors– Mechanisms and Therapeutic Potentials.. Pharmacology & Therapeutics.

[50] Arcaroli J, Yum H-K, Kupfner J, Park JS, Yang K-Y (2001). Role of p38 MAP Kinase in the Development of Acute Lung Injury.. Clinical Immunology.

[51] Peng X, Damarla M, Skirball J, Nonas S, Wang X (0000). Protective role of PI3-kinase/Akt/eNOS signaling in mechanical stress through inhibition of p38 mitogen-activated protein kinase in mouse lung.. Acta Pharmacol Sin.

[52] Le A, Damico R, Damarla M, Boueiz A, Pae HH (2008). Alveolar cell apoptosis is dependent on p38 MAP kinase-mediated activation of xanthine oxidoreductase in ventilator-induced lung injury.. Journal of Applied Physiology.

[53] Kang YJ, Seit-Nebi A, Davis RJ, Han J (2006). Multiple Activation Mechanisms of p38α Mitogen-activated Protein Kinase.. Journal of Biological Chemistry.

[54] Dolinay T, Wu W, Kaminski N, Ifedigbo E, Kaynar AM (2008). Mitogen-Activated Protein Kinases Regulate Susceptibility to Ventilator-Induced Lung Injury.. PLoS ONE.

[55] Li L-F, Yu L, Quinn DA (2004). Ventilation-induced neutrophil infiltration depends on c-Jun N-terminal kinase.. Am J Respir Crit Care Med.

[56] Dinarello CA, Schindler R (1990). Dissociation of transcription from translation of human IL-1-beta: the induction of steady state mRNA by adherence or recombinant C5a in the absence of translation.. Prog Clin Biol Res.

[57] Sandler H, Stoecklin G (2008). Control of mRNA decay by phosphorylation of tristetraprolin.. Biochem Soc Trans.

[58] Nick JA, Young SK, Arndt PG, Lieber JG, Suratt BT (2002). Selective suppression of neutrophil accumulation in ongoing pulmonary inflammation by systemic inhibition of p38 mitogen-activated protein kinase.. J Immunol.

