# Supplementary figures and images for: Using the One-Lung Method to Link p38 to Pro-Inflammatory Gene Expression during Overventilation in C57BL/6 and BALB/c Mice
Source: PLoS One. 2012 Jul 24;7(7):e41464. doi: 10.1371/journal.pone.0041464 (PMC3404097; doi:10.1371/journal.pone.0041464)

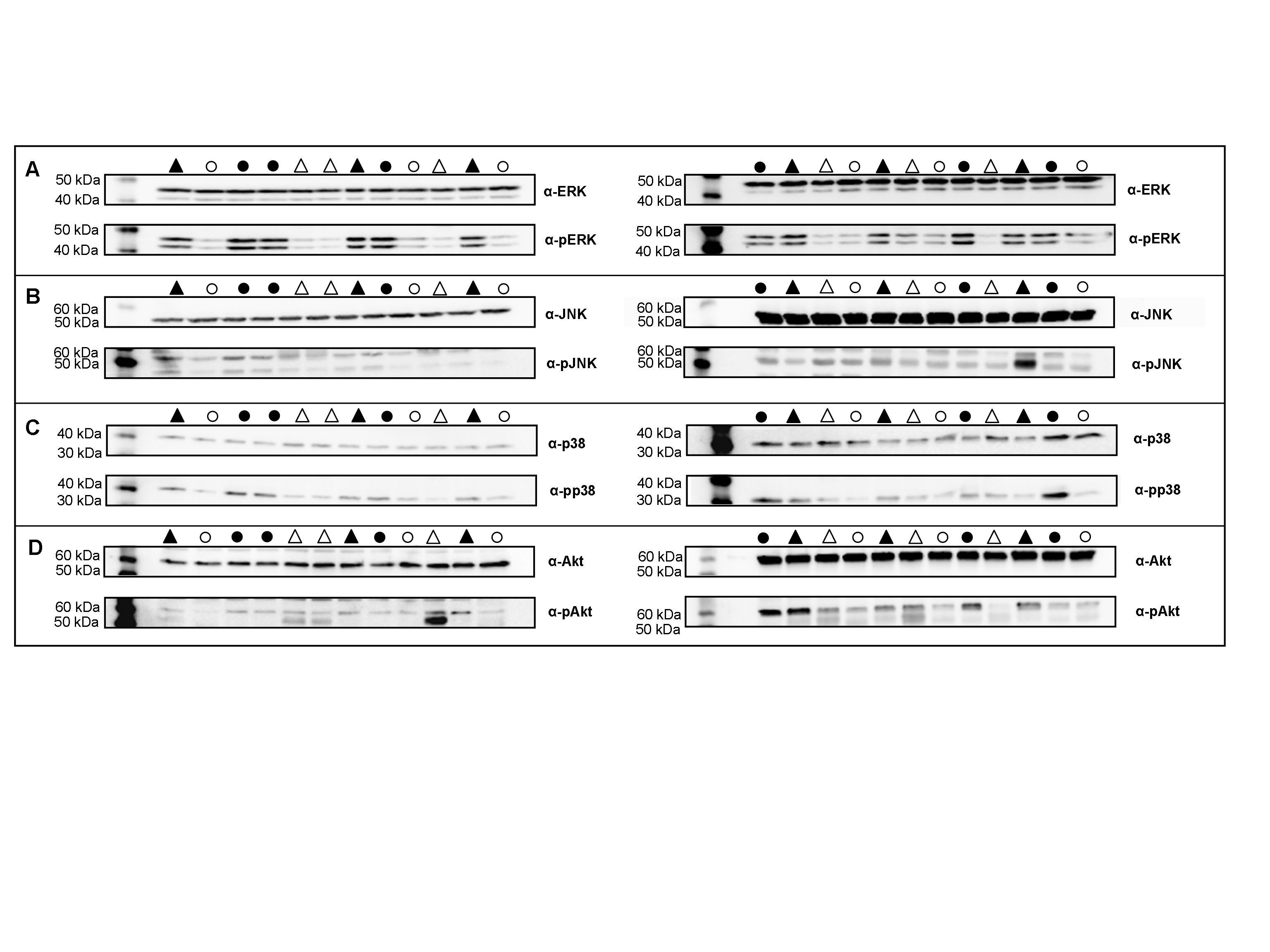

Supplement: Figure S1 — Ventilation and strain-dependent differences in kinase phosphorylation in C57BL/6 and BALB/c mice. Left lungs were harvested after 180 min of ventilation and perfusion and probed for the native and the phosphorylated form of ERK (A), JNK (B), p38 (C) and Akt (D). C57BL/6 = triangles; BALB/c = circles; LPV = open symbols; HPV = closed symbols (TIF) [file pone.0041464.s001.tif]
